# Quantifying the increased risk of illness in malnourished children: a global meta-analysis and propensity score matching approach

**DOI:** 10.1186/s41256-024-00371-0

**Published:** 2024-07-31

**Authors:** Mukhtar A. Ijaiya, Seun Anjorin, Olalekan A. Uthman

**Affiliations:** 1 Jhpiego, Plot 971, Rueben Okoya Crescent, Off Okonjo Iweala Street, Wuye District, Abuja, FCT Nigeria; 2 Big Data Institute, Nuffield Department of Population Health, University of Oxford, Oxford, UK; 3 Warwick Centre for Global Health, Division of Health Sciences, Warwick Medical School, University of Warwick, Coventry, CV4 7AL UK

**Keywords:** Malnutrition, Morbidity, Childhood, Meta-analysis, Propensity score matching

## Abstract

**Background:**

Childhood morbidity and mortality continue to be major public health challenges. Malnutrition increases the risk of morbidity and mortality from illnesses such as acute respiratory infections, diarrhoea, fever, and perinatal conditions in children. This study explored and estimated the magnitude of the associations between childhood malnutrition forms and child morbidity.

**Methods:**

We performed an individual participant data (IPD) meta-analysis and employed propensity score matching to examine crude (unadjusted) and adjusted associations. Our analysis utilized demographic and health datasets from surveys conducted between 2015 and 2020 in 27 low- and middle-income countries. Our objective was to quantify the risk of morbidity in malnourished children and estimate the population-attributable fraction (PAF) using a natural experimental design with a propensity score-matched cohort.

**Results:**

The IPD meta-analysis of child morbidity across three childhood malnutrition forms presented nuanced results. Children with double-burden malnutrition had a 5% greater risk of morbidity, which was not statistically significant. In contrast, wasted children had a 28% greater risk of morbidity. Overweight children exhibited a 29% lower risk of morbidity. Using the matched sample, children with double-burden malnutrition and overweight children had lower morbidity risks (1.7%, RR: 0.983 (95% CI, 0.95 to 1.02) and 20%, RR: 0.80 (95% CI, 0.76 to 0.85), respectively), while wasting was associated with a 1.1 times (RR: 1.094 (95% CI, 1.05 to 1.14)) greater risk of morbidity. Eliminating double-burden malnutrition and wasting in the four and seven countries with significant positive risk differences could reduce the child morbidity burden by an estimated average of 2.8% and 3.7%, respectively.

**Conclusions:**

Our study revealed a correlation between specific childhood malnutrition subtypes—double-burden malnutrition and wasting—and increased risks of morbidity. Conversely, overweight children exhibited a lower risk of immediate morbidity, yet they may face potential long-term health challenges, indicating the necessity for nuanced approaches to childhood nutrition.

**Supplementary Information:**

The online version contains supplementary material available at 10.1186/s41256-024-00371-0.

## Background

Childhood morbidity and mortality remain significant concerns in public health discourse despite decades of intervention efforts. As of 2021, global progress has been made in reducing the under-5 mortality rate to 38 per 1,000 live births, decreasing from 12.8 million deaths in 1990 to 5 million deaths in 2021 [1]. However, low- and middle-income countries (LMICs), particularly in sub-Saharan Africa and southern Asia, still contributed to more than 80% of under-5 deaths in 2021 [1–3]. An estimated 45% of these global deaths of children aged under 5 years were attributed to nutrition-related problems [4].

Childhood malnutrition primarily manifests in three distinct forms—stunting, wasting, and overweight/obesity—collectively known as triple threats [5]. Stunting results from poor nutrition or limited access to food, while wasting is a life-threatening condition characterized by a rapid decline in body mass and nutritional status, usually resulting from acute food shortage or illness [5, 6]. Childhood overweight/obesity results from an imbalance in energy consumed and expended and multifaceted interactions of genetic, biological, environmental, economic, and cultural factors [5–7]. These malnutrition forms can be broadly categorized as undernutrition (stunting and wasting) and overweight/obesity.

The double burden of malnutrition, defined as the contrasting coexistence of undernutrition and overnutrition, has become increasingly prevalent. Three key drivers contribute to this childhood malnutrition phenomenon: individual factors, such as maternal and perinatal diets; environmental factors, including diet imbalances, breastfeeding norms, hygiene practices, and clean water access; and socioeconomic factors, such as food insecurity and low food and health literacy levels [8, 9].

Anthropometric variables such as weight, height, sex, and age have been used to create anthropometric indices for assessing children's nutritional status [10]. These indices are expressed in percentiles or z scores and compared to reference values from a healthy population, known as anthropometric standards, to determine nutritional status [10]. Anthropometry is widely favoured for nutritional evaluation due to its noninvasive nature, affordability, and reliable results [11].

Malnourished children face an elevated risk of morbidity and mortality from childhood illnesses such as acute respiratory infections (ARIs), diarrhoea, fever, malaria, and perinatal conditions [1, 4, 12, 13]. In addition, numerous studies have noted that children aged under 5 years who have malnutrition are at a heightened risk of acute and severe childhood illnesses [14–18]. ARIs, diarrhoea, and fever account for a significant proportion of the child morbidity and mortality burdens [19].

Moreover, adults who experience childhood malnutrition may face cognitive impairments and neurodevelopmental and functional deficits, including learning difficulties, low intelligence quotients, and behavioural problems [20, 21]. High malnutrition burdens are closely associated with reduced economic output, a heightened occurrence of infectious and parasitic illnesses leading to physical disabilities, and an increased likelihood of chronic health conditions in adulthood, which contributes to a generational cycle of poverty and poor health, resulting in increased economic losses due to higher medical expenses and other indirect costs [22–25].

Our study aimed to explore and determine the magnitude of the associations between three different forms of childhood malnutrition—wasting, overweight, and the double burden of childhood malnutrition—and child morbidity.

## Methods

### Study design and data sources

This study was based on secondary datasets from recent Demographic and Health Surveys (DHSs) conducted by ICF International in 27 countries between 2015 and 2020 [26]. DHS surveys are household surveys that collect nationally representative data on demographic, environmental, socioeconomic, nutritional, and health indicators from approximately 90 low- and middle-income countries (LMICs) every five years. These high-response rate cross-sectional surveys are conducted using survey methodologies standardized across countries. The surveys follow a stratified multistage cluster sample design to collect data from women and men aged between 15 and 49 years and their young children aged under five years living in randomly selected households from clusters (census enumeration areas) that serve as the primary sampling unit [26]. Children from the DHS datasets of 27 countries, comprising 138,782 mother–child pairs, were included in this study.

### Exposure variables

This study used the World Health Organization (WHO) weight-for-height child growth standard to determine the exposure variables [10]. We selected the weight-for-height Z score as the anthropometric indicator due to its comparative robustness [27]. This standard is based on weight and height measurements expressed as Z scores [10]. Scores below -2 standard deviations from the median indicate moderate or severe wasting, while those above + 2 standard deviations indicate overweight [10]. Therefore, we created two factor variables with two categories each: not wasted and wasted, and not overweight and overweight. In addition, we created a third variable, the double-burden childhood malnutrition variable, with two categories: malnourished (wasted or overweight) and not malnourished. We excluded children with missing or flagged weight-for-height Z scores from our groups.

### Outcome measure

The outcome variable for this study was child morbidity. Child morbidity was defined as having had a fever, an episode of diarrhoea, or symptoms of acute respiratory infection in the two weeks preceding the survey, which are three common childhood conditions [19]. Children exhibiting symptoms of acute respiratory infection were defined as those experiencing short, rapid breathing and/or chest-related breathing difficulties in the two weeks preceding the survey [26]. Consequently, we created a factor variable for the outcome with two categories: no morbidity and morbidity.

### Covariates

Child and maternal covariates included in the analysis were child age, child sex, breastfeeding status, place of residence, pregnancy type, maternal age, maternal education level, maternal employment status, maternal marital status, maternal health behaviour, and household wealth index. Maternal health behaviour was calculated through principal component analysis (PCA) of three factors: maternal knowledge of oral rehydration salts (ORSs), the place of delivery, and child immunization records. Wealth index quintiles, also calculated through PCA, are proxy measures derived from asset ownership [28]. Principal component analysis (PCA) aggregates multiple related variables into components to represent an underlying construct that is otherwise directly unmeasurable [29]. Our covariates were selected based on previous research, availability, and conceptual reasoning [30–32]

### Statistical analysis

In this study, we performed descriptive analysis, individual participant data (IPD) meta-analysis, propensity score matching, and population attributable fraction (PAF) estimation. IPD meta-analysis is considered the gold standard for estimating precise estimates with good statistical power when examining associations between subgroups of participants while accounting for country-study differences [33, 34]. Propensity score matching effectively reduces bias and mitigates imbalances among measured confounders when estimating treatment effects in nonexperimental studies [35]. The PAF estimates the public health impact of childhood malnutrition and the relative child morbidity burden across countries [36, 37]. PAFs are based on perfect interventions that eliminate childhood malnutrition and on the assumption that childhood malnutrition has a linear relationship with child morbidity with no interconnectedness with other risk factors [37, 38]. PAFs should neither be considered the relative strength of the association nor causality but rather the relationship between variables [36, 39].

For descriptive analysis, we examined the distribution of variables by presenting the absolute number (percentages) for categorical variables and the mean (standard deviation, SD) for continuous variables. The analysis was adjusted for selection probabilities using sampling weights.

In the IPD meta-analysis, we calculated crude and covariate-adjusted risk differences (RDs) to examine the association between the exposure variables and child morbidity. We employed a random-effects model using the restricted maximum likelihood (REML) estimation technique to calculate the pooled RD [40]. This accounts for effect heterogeneity and covariate inclusion in evaluating overall treatment efficacy [40]. The homogeneity of the results was assessed using Cochran's Q test. I^2^ was used to quantify the percentage of variation among different studies that contributed to the heterogeneity, with higher values indicating greater heterogeneity [41, 42].

We applied propensity score matching to minimize potential biases and account for differences in baseline characteristics. We reviewed the baseline characteristics of the children and estimated the standardized differences for all variables pre- and postmatching, with a difference >  = 10% indicating imbalance [43]. The propensity score was calculated using a covariate-adjusted logistic regression model, with each malnourished/wasted/overweight child matched with the closest propensity score at a ratio of 1:5 using the nearest neighbour algorithm with no replacement. We examined the matching quality and conducted a comparative descriptive analysis of the matched and unmatched data. In addition, we calculated the average treatment effect of wasting, overweight, and double-burden childhood malnutrition on child morbidity and the difference in the probability of child morbidity in the propensity score-matched cohort.

Using the matched dataset, we estimated the average and individual PAF (and 95% CIs) for each country with a positive significant risk difference from our adjusted IPD meta-analysis using the STATA module punaf, employing logistic regression results [44].

The null hypothesis was tested against a two-sided alternative hypothesis at a 5% significance level. All the analyses were performed using STATA 16 [45].

## Results

### General demographic and health surveys data by country

The analyses involved 138,782 children, ranging from 1,082 children in South Africa to 12,033 children in Benin (Table 1). Of the 138,782 children included in this analysis, 6.3% were wasted, and 4.3% were overweight, with a combined double burden of malnutrition incidence of 10.5% (Table 1). Timor Leste had the highest prevalence of wasting (24.2%) and double-burden malnutrition (29.5%) (Table 1). Rwanda (1.2%) and Burundi (6.5%) had the lowest prevalence of wasting and double-burden malnutrition, respectively. Nepal had the lowest proportion of overweight children at 1.3%, while Albania had the highest at 16.9%. The prevalence of child morbidity was 30.2%, ranging from 11.5% in Armenia to 51.8% in Burundi (Table 1). The majority (19) of the countries had a child morbidity prevalence higher than the combined average from our results.

**Table 1:** Description of demographic and health survey data by country

Continent	Country	Survey Year	Number of Children	Number of Clusters	Wasted (%)	Overweight (%)	Double Burden (%)	Morbidity (%)
Europe	Albania	2018	2,462	631	1.4	16.9	18.3	12.8
Africa	Angola	2016	6,407	625	5.0	3.6	8.6	27.3
Asia	Armenia	2016	1,561	304	4.2	13.5	17.7	11.5
Africa	Benin	2018	12,033	555	5.1	2.0	7	26.4
Africa	Burundi	2017	6,052	554	5.1	1.4	6.5	51.8
Africa	Cameroon	2019	4,477	428	4.4	11.1	15.5	24.7
Africa	Gambia	2020	3,811	279	5.3	2.3	7.5	31.6
Africa	Guinea	2018	3,430	399	9.1	6.0	15.1	26.2
Americas	Haiti	2017	5,598	449	3.8	3.6	7.4	48.9
Africa	Liberia	2020	2,457	324	3.7	4.5	8.2	36.0
Africa	Malawi	2016	5,178	850	2.8	4.5	7.3	43.3
Asia	Maldives	2017	2,362	260	9.2	4.1	13.3	26.8
Africa	Mali	2018	8,588	345	8.9	2.0	10.9	27.5
Asia	Nepal	2016	2,369	375	9.8	1.3	11	25.5
Africa	Nigeria	2018	11,405	1,378	6.9	2.1	9	31.7
Asia	Pakistan	2018	4,151	554	7.0	2.5	9.5	50.0
Oceania	Papua New Guinea	2018	3,290	674	9.2	9.0	18.2	28.2
Africa	Rwanda	2020	3,809	500	1.2	5.8	6.9	27.8
Africa	Senegal	2019	5,531	214	8.0	2.4	10.4	24.3
Africa	Sierra Leone	2019	4,144	564	5.6	4.9	10.5	22.3
Africa	South Africa	2016	1,082	466	2.5	13.7	16.2	29.4
Asia	Tajikistan	2017	5,867	366	5.5	3.3	8.8	18.4
Africa	Tanzania	2016	8,962	607	4.8	3.8	8.5	27.1
Asia	Timor-Leste	2016	5,718	455	24.2	5.4	29.5	19.0
Africa	Uganda	2016	4,413	688	3.8	4.0	7.8	50.0
Africa	Zambia	2019	8,711	545	4.3	5.3	9.6	26.7
Africa	Zimbabwe	2015	4,914	399	3.5	5.9	9.4	30.0
Total			**138,782**	**13,788**	**6.3**	**4.3**	**10.5**	**30.2**

### Descriptive statistics of covariates

Table 2 provides a descriptive summary of the covariates. The mean age of the population was 28.4 months, with a standard deviation of 17.3. There were slightly more males (50.6%) than females (49.4%). Most children resided in rural areas (67.0%) and were born to employed mothers (58.7%). Almost all the children were singleton births (97.1%) and had mothers who reported being married (90.0%). The maternal age distribution showed that the largest group consisted of mothers aged 25–34 years (50.2%), followed by those aged 15–24 years (26.8%) and 35–49 years (23.0%). Approximately one-third of the mothers had no education (33.5%) and reported breastfeeding their children (37%) during the survey. The distribution of maternal health behaviour was analysed using quantiles. The first quantile represented the group with the least healthy behaviours (35.9%), followed by the second (33.5%), third (18.1%), fourth (4.8%), and fifth quantiles, which represented the highest level of healthy behaviours (7.8%). The wealth index indicated that the largest group fell into the "poorest" category (22.3%), and the smallest group fell into the "richest" class (16.7%).

**Table 2:** Descriptive statistics of the variables included in the analysis

Variables	Overall (*N* = 138,782)
**Child Age in months Mean (SD)**	28.4 (17.3)
**Child Sex**	
Male	69,116 (50.6)
Female	67,589 (49.4)
**Place of Residence**	
Urban	45,119 (33.0)
Rural	91,587 (67.0)
**Pregnancy Type**	
Singleton Pregnancy	132,734 (97.1)
Multiple Pregnancy	3,972 (2.9)
**Currently Breastfeeding**	
No	84,532 (63.0)
Yes	49,715 (37.0)
**Maternal Age (Y)**	
15–24 years	36,601 (26.8)
25–34 years	68,629 (50.2)
35–49 years	31,476 (23.0)
**Maternal Education Level**	
No Education	45,750 (33.5)
Primary	42,928 (31.4)
Secondary	39,992 (29.3)
Higher	8,029 (5.8)
**Maternal Marital Status**	
Never Married	6,530 (4.8)
Married	123,035 (90.0)
Divorced/Widowed/Separated	7,141 (5.2)
**Maternal Employment Status**	
Not Employed	56,486 (41.3)
Employed	80,194 (58.7)
**Wealth Index**	
Poorest	30,506 (22.3)
Poorer	29,057 (21.3)
Middle	27,761 (20.3)
Richer	26,495 (19.4)
Richest	22,885 (16.7)
**Maternal Health Behaviour**	
First Quantile (Least)	47,853 (35.9)
Second Quantile	44,560 (33.5)
Third Quantile	24,083 (18.1)
Fourth Quantile	6,321 (4.8)
Fifth Quantile (Highest)	10,402 (7.8)

Data are presented as the number (%) unless otherwise specified. No. (%) might not add up to the overall total (100%) due to the application of weights and rounding.

## Double burden of malnutrition

**Fig. 1:**
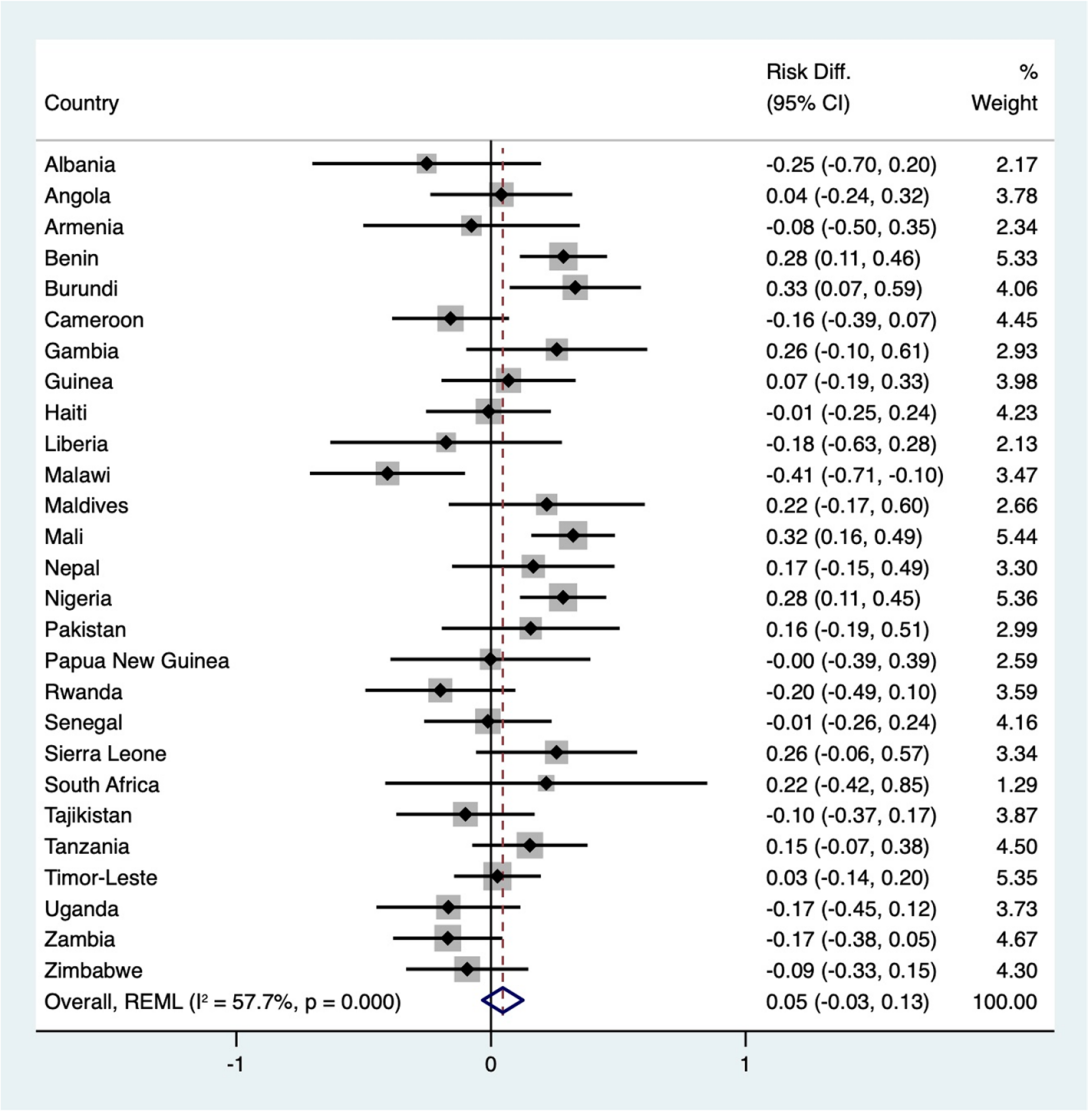
Forest plot of adjusted risk differences for child morbidity among children with double-burden malnutrition compared to non-malnourished children by country. Adjusted for child age, child sex, place of residence, pregnancy type, breastfeeding status, maternal age, maternal education level, maternal marital status, maternal employment status, wealth index, and maternal health behaviour

Figure 1 shows the adjusted risk differences in child morbidity between malnourished and nonmalnourished children. Overall, malnourished children had a 5% greater risk of morbidity, although this difference did not reach statistical significance (95% CI, -0.03 to 0.13). A closer examination by country revealed that four countries—Benin, Burundi, Mali, and Nigeria—had significantly greater risks of morbidity among malnourished children. Interestingly, Malawi, which showed an insignificant negative risk difference in the unadjusted analysis, showed statistical significance in the adjusted analysis. The other twelve countries with negative risk differences in the adjusted analysis did not show statistical significance. Heterogeneity analysis using Cochran's Q test revealed significant variability among the studies (Q value = 61.4, *p* < 0.001), with an I^2^ statistic of 57.7%.

The characteristics of the unmatched and matched children are summarized in Table 3. Malnourished children were younger (mean age 23.1 vs. 29.0; *P* < 0.001), more likely to be born from a multiple pregnancy (3.2 vs. 2.9; *P* = 0.01), and less likely to be female (45.1 vs. 50.0; *P* < 0.001). A greater proportion of the participants were currently breastfeeding (50.1 vs. 35.5; *P* < 0.001) and were less likely to live in middle-income households (19.3 vs. 20.4; *P* < 0.01). Mothers of malnourished children were less likely to be aged 35–49 years (21.7 vs. 23.2; *P* < 0.001), less likely to be divorced/widowed/separated (4.9 vs. 5.3; *P* = 0.01), and less likely to be employed (51.8 vs. 59.5; *P* < 0.001).

**Table 3:** Baseline characteristics of children with double-burden malnutrition compared to non-malnourished children before and after propensity score matching

	**Unmatched (*N* = 138,782)**				**Matched (*N* = 49,372)**			
**Variables**	**Not Double Burden Malnourished**	**Double Burden Malnourished**	***P* Value**	**%Bias**	**Not Double Burden Malnourished**	**Double Burden Malnourished**	***P* Value**	**%Bias**
Total	89.5	10.5			80.3	19.7		
**Child Age in Months Mean (SD)**	29.0 (17.1)	23.1 (17.5)	< 0.001	-33.4	26.0 (16.9)	24.7 (17.4)	0.81	-0.3
**Child Sex**								
Male	50.1	54.9			52.8	53.1		
Female	50.0	45.1	< 0.001	-10.6	47.2	47.0	0.85	0.2
**Place of Residence**								
Urban	33.3	32.8			35.3	37.1		
Rural	67.0	67.2	0.43	-0.7	64.7	62.9	0.04	-2.4
**Pregnancy Type**								
Singleton Pregnancy	97.1	96.8			97.3	96.3		
Multiple Pregnancy	2.9	3.2	0.01	2.3	2.7	3.7	0.002	3.7
**Currently Breastfeeding**								
No	64.5	49.9			58.2	55.6		
Yes	35.5	50.1	< 0.001	28.1	41.8	44.4	0.33	-1.2
**Maternal Age (Y)**								
15–24 years	26.6	28.5			27.5	28.5		
25–34 years	50.3	49.8	0.56	0.5	50.4	48.5	0.06	-2.2
35–49 years	23.2	21.7	< 0.001	-4.6	22.1	23.1	0.37	1.0
**Maternal Education Level**								
No Education	33.4	33.9			32.4	31.2		
Primary	31.7	29.0	< 0.001	-7.1	29.1	29.7	0.22	1.4
Secondary	29.1	33.3	< 0.001	3.2	31.6	31.2	0.90	0.1
Higher	5.8	6.8	< 0.001	5.2	6.9	7.9	0.05	2.4
**Maternal Marital Status**								
Never Married	4.8	4.9			4.4	5.5		
Married	90.0	90.3	0.04	1.8	90.9	88.5	< 0.001	-4.6
Divorced/Widowed/Separated	5.3	4.9	0.01	-2.3	4.7	6.1	0.003	3.3
**Maternal Employment Status**								
Not Employed	40.5	48.2			45.9	46.6		
Employed	59.5	51.8	< 0.001	-17.7	54.1	53.4	0.68	-0.5
**Wealth Index**								
Poorest	22.1	23.8			21.0	19.7		
Poorer	21.2	21.7	0.90	-0.1	20.6	20.7	0.43	0.9
Middle	20.4	19.3	< 0.01	-2.6	20.7	20.6	0.98	0.0
Richer	19.5	18.8	0.14	-1.3	20.0	20.9	0.08	2.0
Richest	16.8	16.4	0.79	-0.2	17.7	18.2	0.68	0.5
**Maternal Health Behaviour**								
First Quantile (Least)	36.0	35.7			34.0	31.8		
Second Quantile	33.4	33.7	0.16	1.2	33.4	33.1	0.95	0.1
Third Quantile	17.9	20.0	< 0.001	4.8	20.5	22.1	0.23	1.4
Fourth Quantile	4.7	4.9	0.22	-1.1	5.0	6.0	0.12	1.8
Fifth Quantile (Highest)	8.1	5.8	< 0.001	-8.2	7.1	7.0	0.52	0.7

After matching 49,372 children (9,874 malnourished and 39,498 nonmalnourished), the absolute standardized differences for all variables used for propensity score matching were less than 10%. Using the matched sample, the average treatment effect showed no statistically significant difference in terms of the risk of child morbidity between malnourished and nonmalnourished children (30.0% vs. 30.4%; *p* = 0.31; overall risk 30.3%). Malnourished children were 1.7% less likely to experience child morbidity than nonmalnourished children were (RR 0.983; 95% CI, 0.95 to 1.02).

**Fig. 2:**
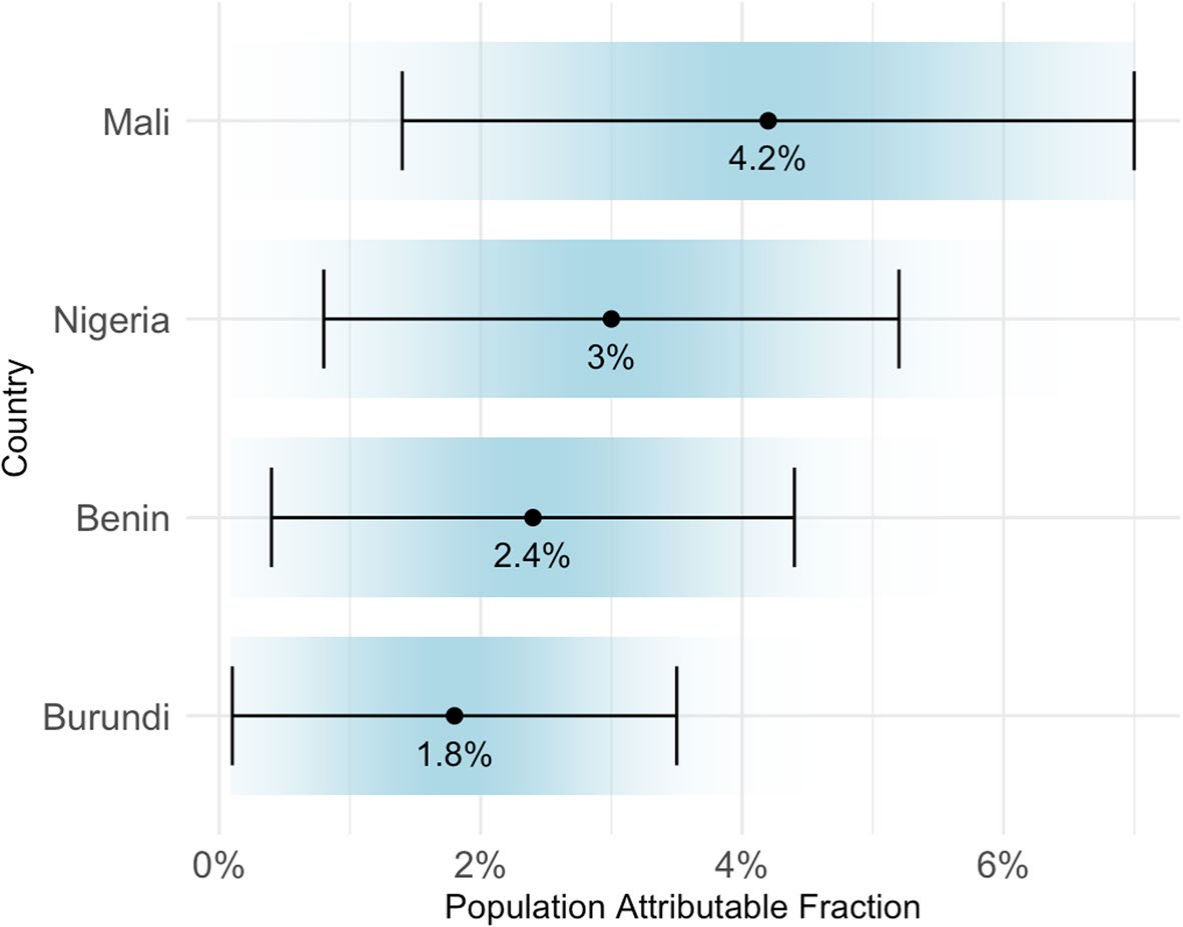
Population-attributable fraction of double-burden malnutrition in child morbidity

An estimated average PAF of 2.8% (95% CI, 1.7% to 3.9%) of the child morbidity burden could be reduced if double-burden childhood malnutrition was eliminated in the four countries with significant positive risk differences. Among these countries, Mali had the highest PAF (4.2%; 95% CI, 1.4% to 7.0%), while Burundi had the lowest PAF (1.8%; 95% CI, 0.1% to 3.5%) (Fig. 2). The PAFs for Nigeria and Benin were 3.0% (95% CI, 0.8% to 5.2%) and 2.4% (95% CI, 0.4% to 4.4%), respectively.

## Wasting

**Fig. 3:**
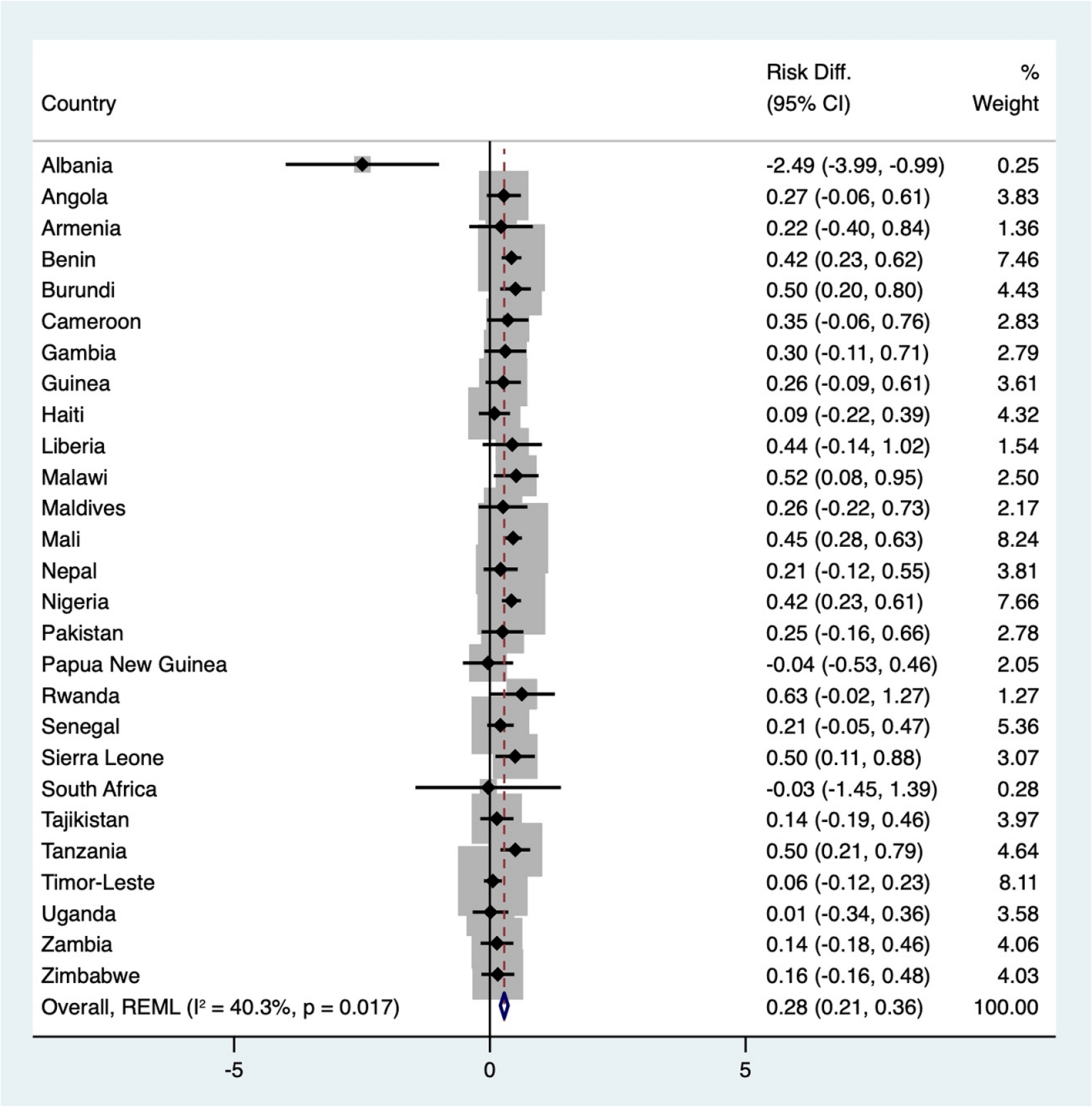
Forest plot of adjusted risk differences for child morbidity between wasted and nonwasted children by country. Adjusted for child age, child sex, place of residence, pregnancy type, breastfeeding status, maternal age, maternal education level, maternal marital status, maternal employment status, wealth index, and maternal health behaviour

Figure 3 shows the differences in the risk of child morbidity between wasted and nonwasted children. The adjusted IPD meta-analysis revealed that overall, wasted children had a 28% greater risk of morbidity (95% CI, 0.21 to 0.36) than nonwasted children. Eight countries showed statistically significant risk differences, including Albania, Benin, Burundi, Malawi, Mali, Nigeria, Sierra Leone, and Tanzania. Only Albania showed a statistically significant negative risk difference. Heterogeneity analysis using Cochran's Q test for the adjusted analysis revealed a Q value of 43.6 (*p* = 0.02) and an I^2^ statistic of 40.3%, indicating moderate variability across studies.

Significant baseline differences existed between wasted and nonwasted children (Table 4). The mean age among the wasted children was lower (24.1 vs. 28.7; *P* < 0.001), and the proportion of wasted female children was lower than that of nonwasted female children (44.6 vs. 49.8; *P* < 0.001). Wasted children were more likely to be born from multiple pregnancies (3.9 vs. 2.8; *P* < 0.001), reside in rural areas (69.9 vs. 66.8; *P* = 0.001), and currently breastfeeding (50.3 vs. 36.1; *P* < 0.001). Children in middle-income countries and the richest households were significantly less likely to be wasted. Mothers of wasted children were less likely to be divorced/widowed/separated (4.5 vs. 5.3; *P* = 0.001) and employed (51.9 vs. 59.1; *P* < 0.001). Significant differences were also observed in maternal health behaviour between mothers of children with and without wasting.

**Table 4:** Baseline characteristics of wasted and non-wasted children before and after propensity score matching

	**Unmatched (*N* = 138,782)**				**Matched (*N* = 35,939)**			
**Variables**	**Not Wasted**	**Wasted**	***P* Value**	**%Bias**	**Not Wasted**	**Wasted**	***P* Value**	**%Bias**
Total	93.7	6.3			81.6	18.4		
**Child Age in Months Mean (SD)**	28.7 (17.2)	24.1 (17.4)	< 0.001	-26.0	25.5 (17.2)	24.8 (17.4)	0.51	-1.0
**Child Sex**								
Male	50.2	55.4			53.1	54.5		
Female	49.8	44.6	< 0.001	-11.0	46.9	45.5	0.27	-1.7
**Place of Residence**								
Urban	33.2	30.1			32.4	33.9		
Rural	66.8	69.9	0.001	3.8	67.6	66.1	0.01	-3.9
**Pregnancy Type**								
Singleton Pregnancy	97.2	96.1			97.0	95.8		
Multiple Pregnancy	2.8	3.9	< 0.001	5.7	3.0	4.2	0.002	4.8
**Currently Breastfeeding**								
No	63.9	49.7			54.4	53.4		
Yes	36.1	50.3	< 0.001	27.6	45.6	46.7	0.83	-0.3
**Maternal Age (Y)**								
15–24 years	26.7	27.2			27.1	28.4		
25–34 years	50.2	49.9	0.19	1.4	51.3	48.2	0.002	-4.7
35–49 years	23.0	23.0	0.08	-2.0	21.6	23.4	0.05	2.9
**Maternal Education Level**								
No Education	33.0	41.0			37.4	36.6		
Primary	31.8	25.9	< 0.001	-14.7	27.4	27.3	0.72	0.5
Secondary	29.3	28.3	0.25	-1.3	29.8	30.6	0.30	1.6
Higher	6.0	4.8	0.002	-3.6	5.4	5.5	0.19	1.9
**Maternal Marital Status**								
Never Married	4.9	3.6			3.3	4.0		
Married	89.9	91.9	< 0.001	6.9	92.5	90.6	0.01	-4.8
Divorced/Widowed/Separated	5.3	4.5	0.001	-3.8	4.2	5.4	0.01	3.5
**Maternal Employment Status**								
Not Employed	40.9	48.1			46.9	47.9		
Employed	59.1	51.9	< 0.001	-16.2	53.1	52.1	0.66	-0.7
**Wealth Index**								
Poorest	22.1	25.8			23.6	21.2		
Poorer	21.2	22.7	0.16	1.6	21.9	22.2	0.39	-1.3
Middle	20.4	18.6	< 0.001	-4.4	19.4	19.9	0.22	1.8
Richer	19.4	18.6	0.37	-1.0	19.2	21.1	0.004	4.3
Richest	16.9	14.3	< 0.001	-5.8	16.0	15.7	0.76	0.5
**Maternal Health Behaviour**								
First Quantile (Least)	35.9	37.0			36.6	33.2		
Second Quantile	33.6	31.4	0.001	-3.6	33.1	31.9	0.83	0.3
Third Quantile	17.8	22.8	< 0.001	12.1	20.9	24.6	0.001	5.2
Fourth Quantile	4.8	3.5	< 0.001	-8.0	3.3	4.2	0.1	2.2
Fifth Quantile (Highest)	8.0	5.4	< 0.001	-10.3	6.3	6.0	0.60	0.7

We successfully matched 35,939 children (10,066 wasted and 40,296 nonwasted). After matching, the absolute standardized differences for all variables used in the propensity score matching were less than 10%. Using the matched sample, the average treatment effect showed that wasted children had a 33.2% risk of child morbidity compared to that of 30.3% among nonwasted children, with an overall risk of 30.9%. Wasted children were 1.1 times more likely to experience child morbidity than nonwasted children were (RR 1.094; 95% CI, 1.05 to 1.14).

**Fig. 4:**
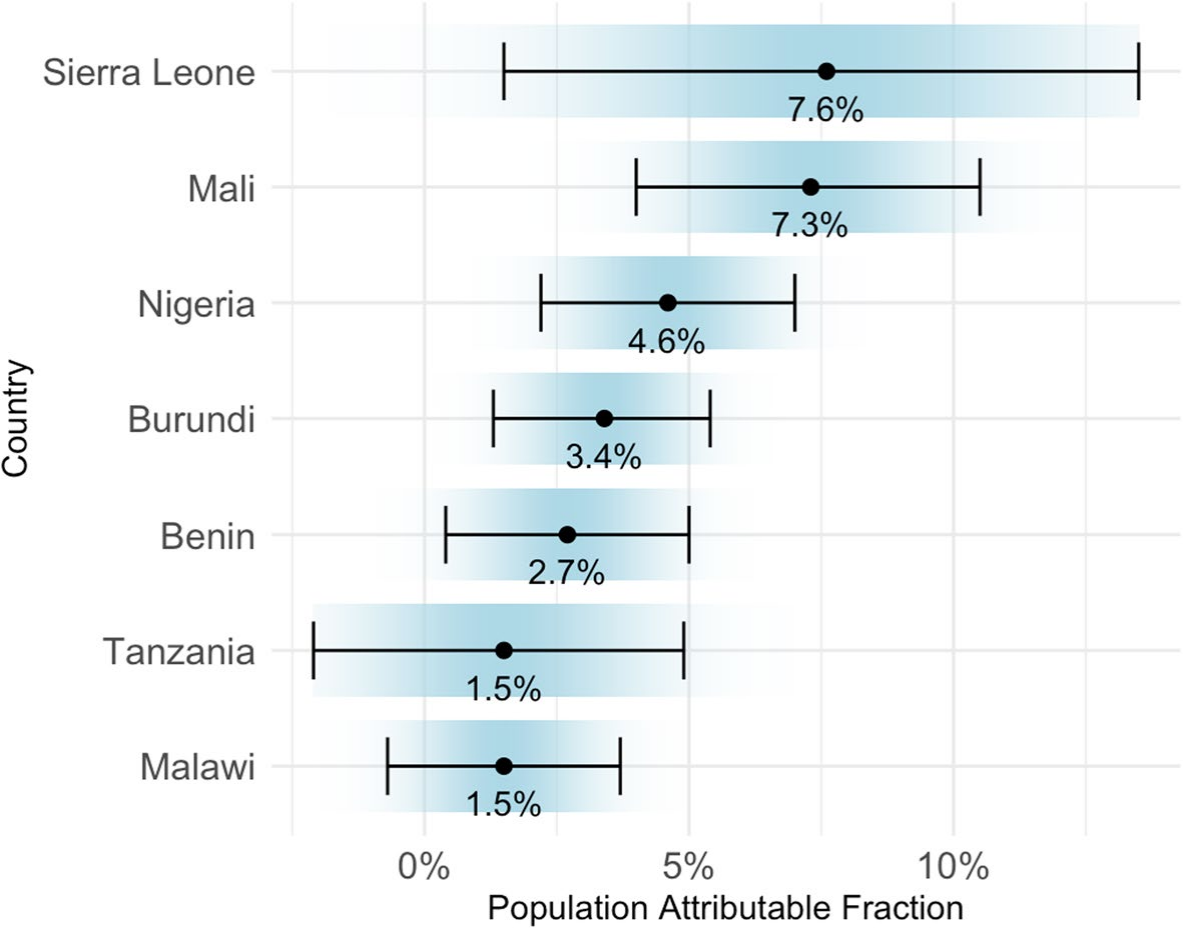
Population attributable fraction of wasting in child morbidity

The estimated average population attributable fraction (PAF) for the seven countries with significant positive risk differences from the adjusted IPD meta-analysis was 3.7% (95% CI, 2.6% to 4.8%). Sierra Leone had the highest PAF estimate at 7.6% (95% CI, 1.5% to 13.5%), while Malawi and Tanzania had the lowest PAF estimates at 1.5% (Malawi: 95% CI, -0.7% to 3.7%; Tanzania: 95% CI, -2.1% to 4.9%) (Fig. 4). By inference, eliminating wasting could reduce the child morbidity burden by 3.7% across these seven countries.

## Overweight

**Fig. 5:**
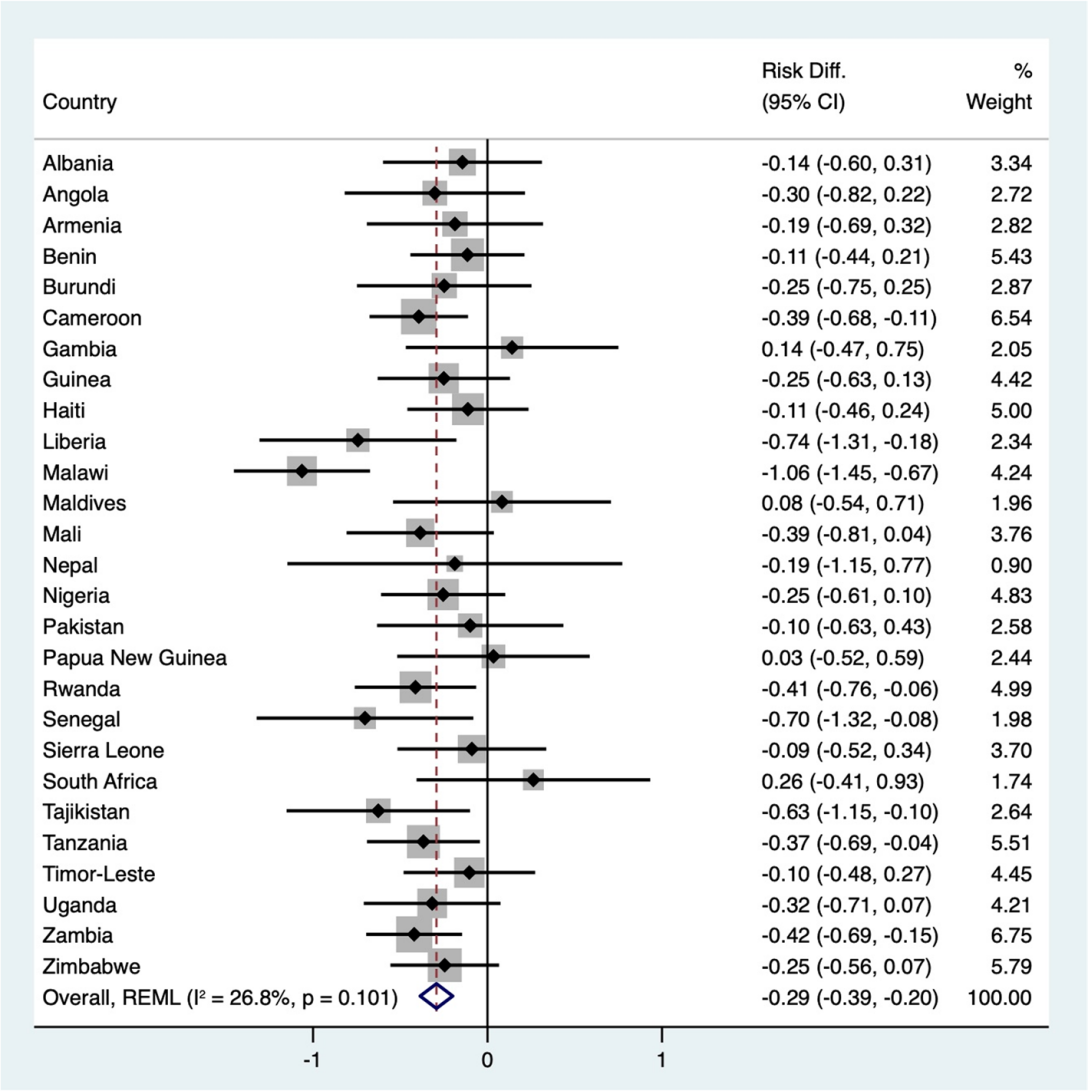
Forest plot of adjusted risk differences for child morbidity between overweight and nonoverweight children by country. Adjusted for child age, child sex, place of residence, pregnancy type, breastfeeding status, maternal age, maternal education level, maternal marital status, maternal employment status, wealth index, and maternal health behaviour

Figure 5 illustrates the differences in the risk of child morbidity between overweight and nonoverweight children. The adjusted IPD meta-analysis showed that overweight children hade an overall 29% lower risk of experiencing morbidity (95% CI, -0.39 to -0.20) than their nonoverweight counterparts. Eight countries, Cameroon, Liberia, Malawi, Rwanda, Senegal, Tajikistan, Tanzania, and Zambia, demonstrated statistically significant negative risk differences. Gambia, Maldives, Papua New Guinea, and South Africa had positive risk differences, although these differences were not statistically significant. Heterogeneity analysis using Cochran's Q test for the adjusted analysis revealed a Q value of 35.5 (*p* = 0.10) and an I^2^ statistic of 26.8%, indicating moderate variability across studies.

The characteristics of the unmatched and matched children are summarized in Table 5. Significant baseline differences were observed between overweight and nonoverweight children. Overweight children were younger (mean age 21.7 vs. 28.7; *P* < 0.001), less likely to be female (45.9 vs. 49.6; *P* < 0.001), less likely to reside in rural areas (63.3 vs. 67.2; *P* < 0.001), and more likely to be born from multiple pregnancies (2.2 vs. 2.9; *P* = 0.01). A greater proportion of overweight children were currently breastfeeding (49.8 vs. 36.5; *P* < 0.001). Mothers of overweight children were less likely to be aged 35–49 years (19.9 vs. 23.2; *P* < 0.001), married (87.8 vs. 90.1; *P* < 0.001), and employed (51.6 vs. 59.0; *P* < 0.001). Children from the richest households were significantly more likely to be overweight (19.5 vs. 16.6; *P* < 0.001).

**Table 5:** Baseline characteristics of overweight and nonoverweight children before and after propensity score matching

	**Unmatched (*N* = 138,782)**				**Matched (*N* = 25,406)**			
**Variables**	**Not Overweight**	**Overweight**	***P *Value**	**%Bias**	**Not Overweight**	**Overweight**	***P* Value**	**%Bias**
Total	95.8	4.3			81.8	18.2		
**Child Age in Months Mean (SD)**	28.7 (17.2)	21.7 (17.5)	< 0.001	-39.9	23.4 (17.0)	22.6 (17.3)	0.62	0.9
**Child Sex**								
Male	50.4	54.2			53.6	53.2		
Female	49.6	45.9	< 0.001	-8.5	46.4	46.8	0.95	0.1
**Place of Residence**								
Urban	32.8	36.7			36.6	39.0		
Rural	67.2	63.3	< 0.001	-7.4	63.4	61.0	0.23	-2.3
**Pregnancy Type**								
Singleton Pregnancy	97.1	97.8			97.8	97.9		
Multiple Pregnancy	2.9	2.2	0.01	-3.8	2.2	2.1	0.26	2.0
**Currently Breastfeeding**								
No	63.5	50.2			55.2	53.6		
Yes	36.5	49.8	< 0.001	24.9	44.8	46.4	0.50	-1.3
**Maternal Age (Y)**								
15–24 years	26.6	30.5			29.2	29.7		
25–34 years	50.2	49.7	0.49	-0.9	50.8	49.1	0.17	-2.6
35–49 years	23.2	19.9	< 0.001	-8.1	20.0	21.3	0.06	3.5
**Maternal Education Level**								
No Education	33.9	23.2			25.4	24.2		
Primary	31.3	33.6	< 0.001	4.8	32.3	32.7	0.83	-0.4
Secondary	29.1	33.3	< 0.001	9.5	32.8	32.6	0.95	0.1
Higher	5.7	9.8	< 0.001	15.9	9.6	10.5	0.42	1.7
**Maternal Marital Status**								
Never Married	4.7	6.8			5.3	6.6		
Married	90.1	87.8	< 0.001	-5.6	90.4	87.4	0.003	-5.6
Divorced/Widowed/Separated	5.2	5.4	0.90	0.2	4.4	6.0	0.02	4.3
**Maternal Employment Status**								
Not Employed	41.0	48.4			48.2	46.5		
Employed	59.0	51.6	< 0.001	-17.6	51.8	53.5	0.45	1.4
**Wealth Index**								
Poorest	22.4	20.8			20.6	19.4		
Poorer	21.3	20.3	0.05	-2.7	20.3	19.9	0.64	-0.9
Middle	20.3	20.3	0.82	0.3	20.3	20.6	0.79	0.5
Richer	19.4	19.1	0.23	-1.6	18.1	20.1	0.20	2.4
Richest	16.6	19.5	< 0.001	7.9	20.5	20.1	0.90	-0.3
**Maternal Health Behaviour**								
First Quantile (Least)	36.0	33.7			35.5	32.5		
Second Quantile	33.3	37.1	< 0.001	8.3	36.2	36.9	0.93	-0.2
Third Quantile	18.2	15.8	< 0.001	-7.8	15.7	16.4	0.26	2.0
Fourth Quantile	4.7	6.9	< 0.001	8.1	5.6	7.2	0.10	3.3
Fifth Quantile (Highest)	7.9	6.5	0.003	-4.2	57.1	7.0	0.57	1.0

We successfully matched 25,406 children (4,610 overweight and 20,796 nonoverweight). After matching, the absolute standardized differences for all variables used in the propensity score matching were less than 10%. Using the matched sample, the average treatment effect showed that overweight children had a 24.4% risk of child morbidity compared to that of 30.4% among nonoverweight children, with an overall risk of 29.3%. Overweight children were 20% less likely to experience child morbidity than nonoverweight children were (RR 0.800; 95% CI, 0.76 to 0.85).

We did not estimate the PAF because all the countries with statistical significance had a negative risk difference according to our adjusted meta-analysis, indicating a protective effect of overweight against child morbidity.

## Discussion

The prevalence of double-burden malnutrition, wasting, overweight, and child morbidity varied across the studied countries. Our analysis of 138,782 children across 27 countries revealed that 6.3% were wasted and 4.3% were overweight, leading to a combined 10.5% prevalence of the double burden of malnutrition. Child morbidity was reported at 30.2%, with significant variations ranging from 11.5% in Armenia to 51.8% in Burundi.

The adjusted IPD meta-analysis data indicated that double-burden malnourished children had a statistically nonsignificant 5% greater risk of morbidity. After propensity score matching, double-burdened malnourished children were 1.7% less likely to experience morbidity, although the difference was not statistically significant. Eliminating double-burden childhood malnutrition in the four countries with significant positive risk differences could reduce the child morbidity burden by an estimated average of 2.8%.

Wasted children were found to be at 28% greater risk of morbidity in our adjusted meta-analysis. After propensity score matching, wasted children were at a 1.1 times greater risk of morbidity. According to our estimation, 3.7% of the child morbidity burden could be reduced if wasting is eliminated in the seven countries with significant positive risk differences. Our adjusted meta-analysis also revealed that overweight children were at 29% lower risk of morbidity. After propensity score matching, overweight children had a 20.0% lower risk of morbidity.

Similar to our findings reported above, multiple studies have noted increased child morbidity and mortality risk among wasted and double-burdened malnourished children [46–49]. Previous studies have reported the synergistic effect of nutrition, particularly undernutrition and morbidity, among children, suggesting that undernourished children are more prone to infections and morbidities, reducing appetite and limiting nutrient assimilation [46, 50]. This "vicious cycle" between undernutrition and infections has been reported in multiple studies [51–54]. There are various explanations for this cyclical relationship between childhood undernutrition and morbidity.

Childhood undernutrition has been observed to have an effect on innate and adaptive immune functions, causing dysfunction of the immune response and increased susceptibility to infections [55, 56]. Furthermore, intestinal structure and functional changes due to undernutrition and consequent metabolic reactions have been associated with poor growth, development, and dysregulated immune function in children [55–57]. Interestingly, feeding and dietary factors have been found to be crucial factors for severe illness and death [58]. Exclusively breastfed children have been found to have a lower risk of morbidity, with a larger proportion of children at risk of illness in the second six months of life than in the first six months [59–61].

The later manifestation of chronic morbidity in adults who were overweight in childhood may explain our findings that child morbidity risk differs between overweight and nonoverweight children. In previous studies, overweight children have been found to be at increased risk of psychological morbidity and the presence and clustering of cardiometabolic risk factors in childhood [62, 63]. Being overweight may persist until adulthood and is predictive of the development of chronic cardiometabolic morbidities and premature mortality in adults [62, 64, 65]. More specifically, overweight children are at greater risk of accelerated vascular ageing and disease, type 2 diabetes, fatty liver disease, and dyslipidaemia [66, 67]. Notably, Shibli et al., 2007 reported less-than-expected hospital admissions and repeated admissions among overweight infants [68]. Furthermore, in LMICs, being overweight is often perceived as a sign of wealth and is more prevalent among higher-income households that generally have better social determinants of health [69].

Chang et al., 2015 highlighted that overweight-related hypertension may be linked to low-grade inflammation and endothelial dysfunction, while also noting the association between serum cholesterol ratios and coronary artery disease outcomes [70]. Additionally, there is increasing evidence that elevated biomarkers of inflammation in overweight children correlate with the risk of developing type 2 diabetes [70].

In addition to malnutrition, several other factors have been identified as determinants of childhood morbidity, including socioeconomic status, place of residence, maternal occupational status, maternal education level, birth interval, and autonomy [48, 59, 60, 71–73]. These social determinants of health have also been found to be key determinants of childhood malnutrition in multiple studies, further supporting the synergistic relationship between morbidity and malnutrition [71, 74]. The changes in our effect sizes and heterogeneity values pre- and postadjustment further highlight the associations among our included covariates, childhood malnutrition, and child morbidity. Recent studies have also identified the harmful effects of COVID-19-related food, health, and economic disruptions on childhood malnutrition and morbidity. COVID-19 has been shown to increase the prevalence of childhood malnutrition by 14.3%, potentially leading to 128,605 additional under-5 deaths [75, 76].

In our adjusted meta-analysis results regarding the three forms of malnutrition, Malawi had a significant negative risk difference for double-burden malnutrition and overweight and a significant positive risk difference for wasting. Overweight children accounted for approximately 60% of the country’s double burden of malnutrition. In Albania, the only country with a significant negative risk difference in the adjusted meta-analysis for wasting, 16.9% of children were overweight compared to just 1.4% of children with wasting, accounting for 92%-8% of the country’s double of burden malnutrition. Benin, Burundi, Mali, and Nigeria had significant positive risk differences in the adjusted meta-analysis for both double-burden malnutrition and wasting. They all had more wasted children than overweight children. Tanzania, with an almost equal proportion of both wasted (4.8%) and overweight (3.8%) children, showed a significant positive risk difference among wasted children and a significant negative risk difference among overweight children.

The variation in the effect sizes before and after adjustment suggested a moderate level of heterogeneity between countries. Therefore, the magnitude of the risk difference in each country across the different forms of malnutrition and the pooled estimates varied due to each country's differing characteristics and peculiarities. The majority of countries with significant differences in the risk of child morbidity across the three forms of malnutrition examined were in or bordering the West and East Africa subregions. Previous findings from two meta-analyses on childhood malnutrition noted the disproportionate vulnerability of children in the West and East African regions [77, 78]. Moreover, apart from Albania, a country with a high human development index, and Tajikistan and Zambia, countries with a medium human development index, all countries with significant differences had a low human development index [79].

Comprehensive policy initiatives are needed to enhance child nutrition and health outcomes. Given the connection between the double burden of malnutrition, wasting, and increased child morbidity in some countries, it is crucial that policy-makers focus on both nutrition-specific and nutrition-sensitive interventions. Nutrition-specific actions, such as micronutrient supplementation, tailored feeding practices for infants and young children, and effective management of severe malnutrition, directly address nutritional deficits [80, 81]. Concurrently, investment in nutrition-sensitive strategies, including agricultural improvements, social safety nets, and early childhood development programs, can indirectly bolster nutrition by enhancing overall living conditions and food security within communities [80, 81]. Although our study revealed that overweight children exhibited a lower risk of immediate morbidity, they may face potential long-term cardiometabolic and health challenges, indicating the need for nuanced approaches to childhood nutrition that address both immediate and future health risks.

Additionally, our adjusted meta-analyses highlight the important role of social determinants of health, including socioeconomic status, education, and living environment, in shaping child health outcomes. Therefore, effective policies should focus on nutritional interventions and engage broader socioeconomic strategies aimed at poverty alleviation, educational enhancements, and improved living conditions [82, 83]. By addressing these social determinants, policy-makers can develop a more holistic approach to reducing child morbidity.

This study has considerable strengths, such as the diverse sample size and robust statistical techniques used, but it also has limitations, including the correlational nature and potential recall bias. The use of data from 27 countries provided a broad and diverse sample, providing considerable statistical power and improving the generalizability of the results. Furthermore, our application of propensity score matching techniques enabled more precise estimates of the effect of malnutrition on child morbidity, enhancing the study's internal validity [84].

Nevertheless, our findings are correlational and based on cross-sectional data; hence, causal relationships cannot be established [85]. Recall bias might also have affected the results, as our data were obtained through maternal recall, which can be affected by memory and interpretation errors [86]. Additionally, residual confounding is a potential concern, as not all possible confounding variables may have been included in our models [87]. In addition, propensity score matching, while effective in balancing observed variables, does not account for unobserved or unmeasured confounders [88]. Finally, the generalizability of our findings might be limited to the countries included in this analysis and may not hold for other regions or countries with different socioeconomic or cultural contexts [89].

## Conclusions

Our study highlights the variation in the prevalence of double-burden malnutrition, wasting, overweight, and child morbidity across 27 countries. We found a correlation between specific childhood malnutrition subtypes—double-burden malnutrition and wasting—and increased morbidity risks, as well as a protective but complex role of overweight status in childhood. Reducing double-burden malnutrition and wasting could considerably lower the overall morbidity rates in children, improving health outcomes. Conversely, overweight children exhibit a lower risk of immediate morbidity, yet they may face potential long-term health challenges. Our results indicate the need for targeted and nuanced interventions that address malnutrition subtypes and their associated health outcomes. Policy-makers should prioritize nutrition-specific actions, such as micronutrient supplementation and tailored feeding practices, along with nutrition-sensitive strategies, including improvements in agriculture, social safety nets, and early childhood development programs. Effective policies should integrate broader socioeconomic strategies aimed at poverty alleviation, educational enhancements, and improved living conditions to create a holistic approach to reducing child morbidity.

### Supplementary Information

Additional file 1. Forest Plot of unadjusted Risk Differences for Child Morbidity among Children with Double-Burden Malnutrition Compared to Non-Malnourished Children by Country.

Additional file 2. Forest Plot of unadjusted Risk Differences for Child Morbidity Between Wasted and Nonwasted Children by Country.

Additional file 3. Forest Plot of unadjusted Risk Differences for Child Morbidity between Overweight and Nonoverweight Children by Country.

Additional file 4. Supplementary Tables.

## Data Availability

All data and datasets generated and/or analysed during the current study are available on the DHS program website https://dhsprogram.com/data.
