# Supplementary figures and images for: Quantifying the increased risk of illness in malnourished children: a global meta-analysis and propensity score matching approach
Source: Glob Health Res Policy. 2024 Jul 31;9:29. doi: 10.1186/s41256-024-00371-0 (PMC11290152; doi:10.1186/s41256-024-00371-0)

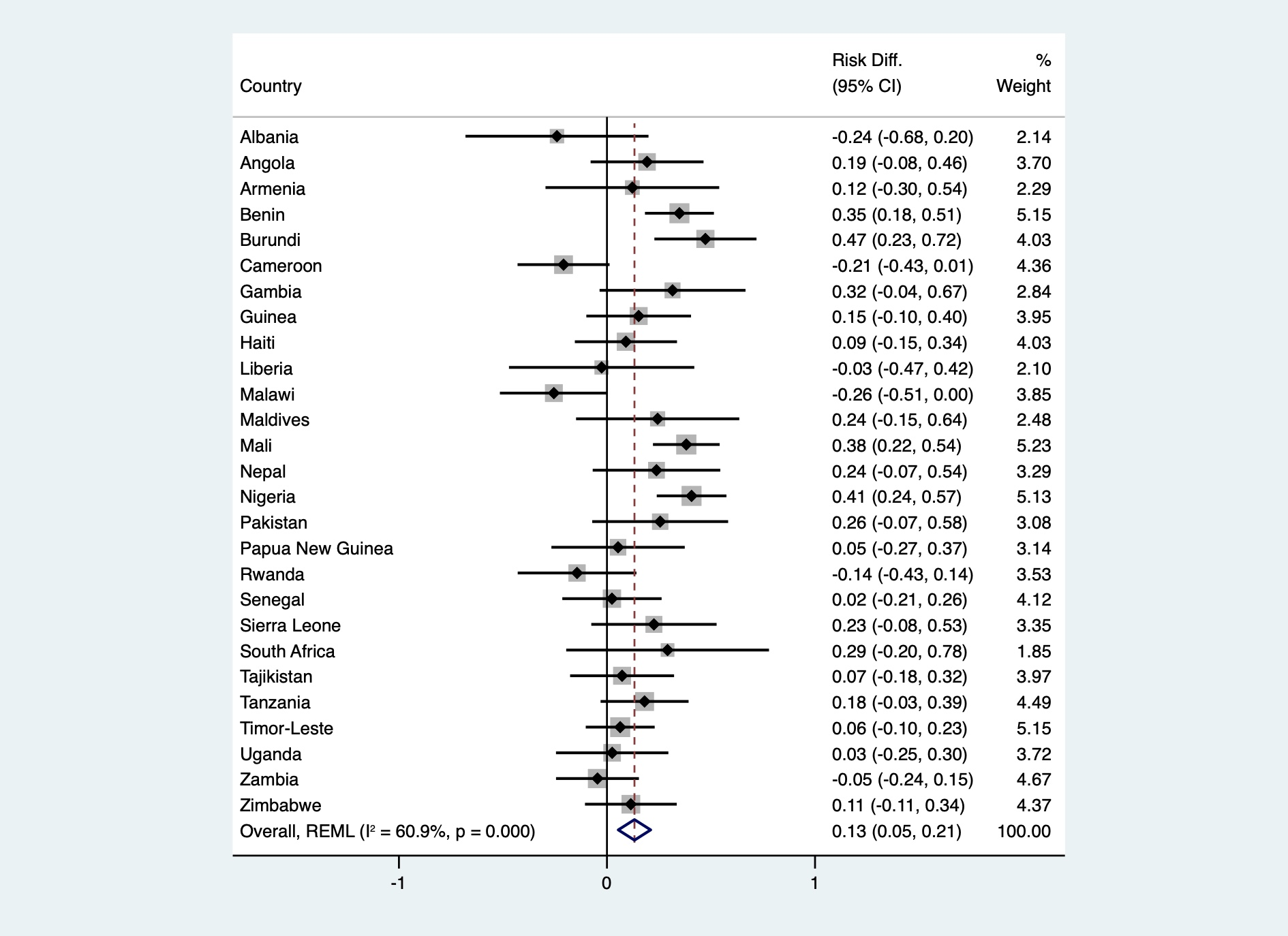

Supplement: Supplementary file 1 — Additional file 1. Forest Plot of unadjusted Risk Differences for Child Morbidity among Children with Double-Burden Malnutrition Compared to Non-Malnourished Children by Country. [file 41256_2024_371_MOESM1_ESM.tif]

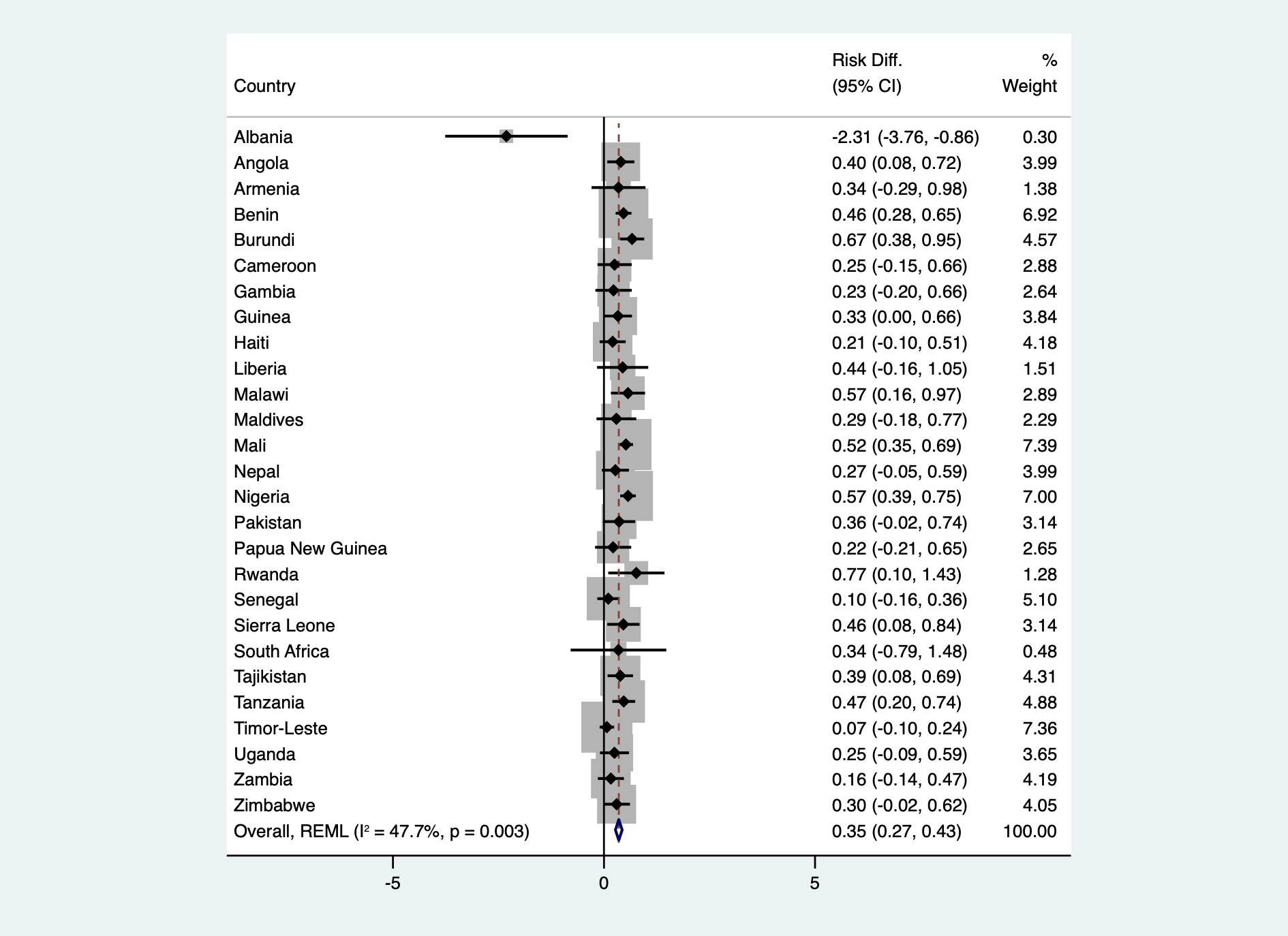

Supplement: Supplementary file 2 — Additional file 2. Forest Plot of unadjusted Risk Differences for Child Morbidity Between Wasted and Nonwasted Children by Country. [file 41256_2024_371_MOESM2_ESM.tif]

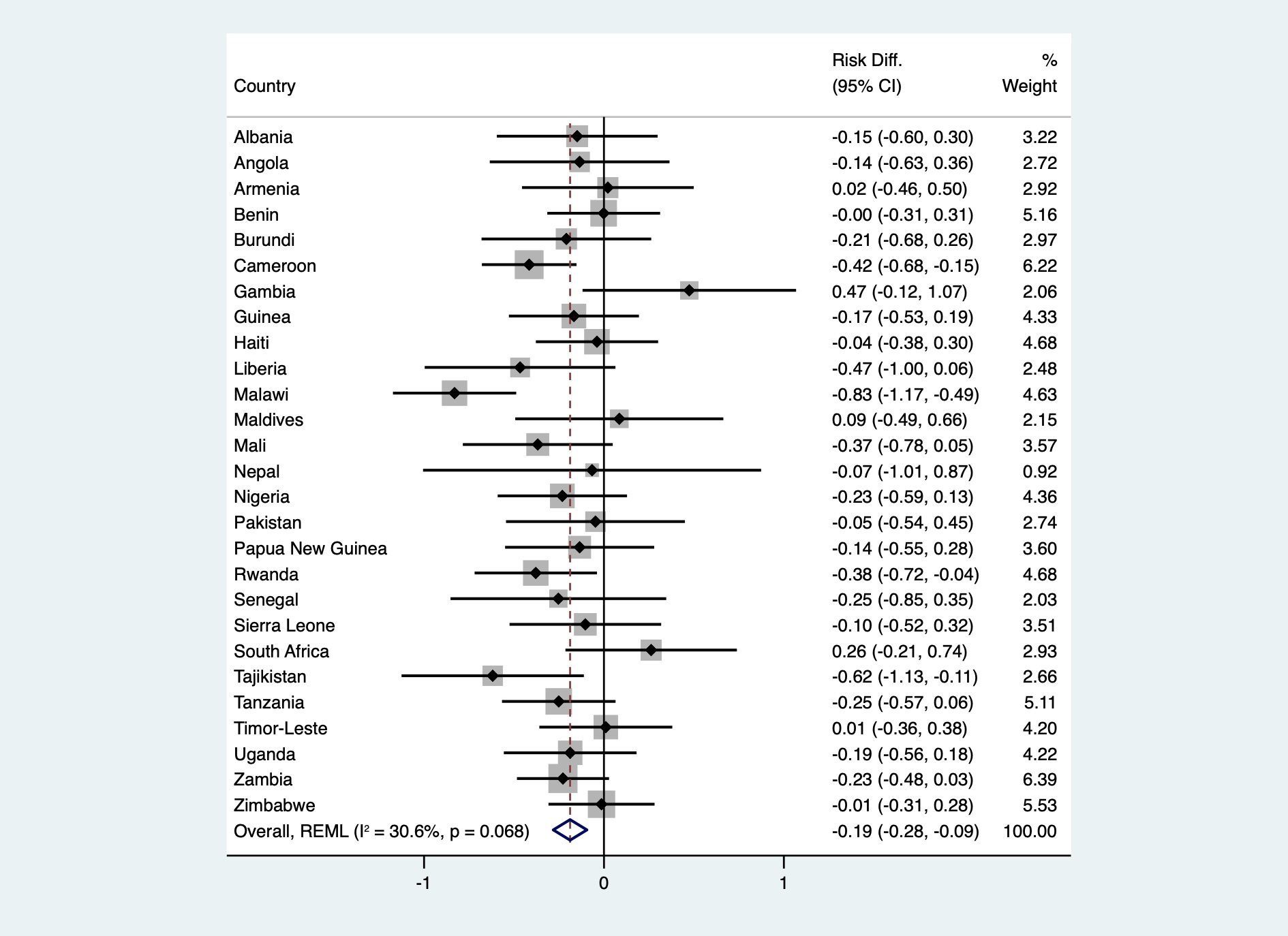

Supplement: Supplementary file 3 — Additional file 3. Forest Plot of unadjusted Risk Differences for Child Morbidity between Overweight and Nonoverweight Children by Country. [file 41256_2024_371_MOESM3_ESM.tif]
