# Supplementary material for: Quantifying the increased risk of illness in malnourished children: a global meta-analysis and propensity score matching approach
Source: Glob Health Res Policy. 2024 Jul 31;9:29. doi: 10.1186/s41256-024-00371-0 (PMC11290152; doi:10.1186/s41256-024-00371-0)
Supplement: Supplementary file 4 — Additional file 4. Supplementary Tables. [file 41256_2024_371_MOESM4_ESM.docx]

**Supplementary Tables**

Table S 1: Unadjusted Risk Differences for Child Morbidity among Children with Double-Burden Malnutrition Compared to Non-Malnourished Children by Country.

| **Country** | **Risk Difference** | **95% Conf. Interval** | | **% Weight** |
| --- | --- | --- | --- | --- |
| Albania | -0.24 | -0.68 | 0.2 | 2.14 |
| Angola | 0.193 | -0.079 | 0.464 | 3.7 |
| Armenia | 0.122 | -0.296 | 0.54 | 2.29 |
| Benin | 0.348 | 0.183 | 0.514 | 5.15 |
| Burundi | 0.473 | 0.228 | 0.719 | 4.03 |
| Cameroon | -0.209 | -0.43 | 0.013 | 4.36 |
| Gambia | 0.315 | -0.036 | 0.666 | 2.84 |
| Guinea | 0.152 | -0.099 | 0.404 | 3.95 |
| Haiti | 0.091 | -0.154 | 0.337 | 4.03 |
| Liberia | -0.026 | -0.471 | 0.42 | 2.1 |
| Malawi | -0.255 | -0.515 | 0.004 | 3.85 |
| Maldives | 0.244 | -0.149 | 0.636 | 2.48 |
| Mali | 0.381 | 0.221 | 0.542 | 5.23 |
| Nepal | 0.238 | -0.069 | 0.545 | 3.29 |
| Nigeria | 0.407 | 0.239 | 0.574 | 5.13 |
| Pakistan | 0.256 | -0.071 | 0.583 | 3.08 |
| Papua New Guinea | 0.053 | -0.267 | 0.374 | 3.14 |
| Rwanda | -0.144 | -0.429 | 0.142 | 3.53 |
| Senegal | 0.024 | -0.215 | 0.263 | 4.12 |
| Sierra Leone | 0.226 | -0.075 | 0.527 | 3.35 |
| South Africa | 0.291 | -0.196 | 0.779 | 1.85 |
| Tajikistan | 0.073 | -0.177 | 0.322 | 3.97 |
| Tanzania | 0.181 | -0.031 | 0.392 | 4.49 |
| Timor-Leste | 0.064 | -0.102 | 0.229 | 5.15 |
| Uganda | 0.025 | -0.245 | 0.295 | 3.72 |
| Zambia | -0.046 | -0.245 | 0.154 | 4.67 |
| Zimbabwe | 0.115 | -0.106 | 0.335 | 4.37 |
| **Overall (p=0.001)** | **0.132** | **0.054** | **0.21** | **100** |
| **Heterogeneity Measures** | **Value** | **df** | | **p-value** |
| Cochran's Q | 66.55 | 26 | | 0.000 |
|  | | **95% Conf. Interval** | |  |
| H | 1.6 | 1.141 | 2.058 |  |
| I2 (%) | 60.9% | 23.2% | 76.4% |  |

Table S 2: Adjusted Risk Differences for Child Morbidity among Children with Double-Burden Malnutrition Compared to Non-Malnourished Children by Country^[[1]](#footnote-1)^.

| **Country** | **Risk Difference** | **95% Conf. Interval** | | **% Weight** |
| --- | --- | --- | --- | --- |
| Albania | -0.252 | -0.702 | 0.197 | 2.17 |
| Angola | 0.041 | -0.238 | 0.319 | 3.78 |
| Armenia | -0.077 | -0.502 | 0.349 | 2.34 |
| Benin | 0.285 | 0.113 | 0.456 | 5.33 |
| Burundi | 0.331 | 0.073 | 0.589 | 4.06 |
| Cameroon | -0.159 | -0.389 | 0.071 | 4.45 |
| Gambia | 0.258 | -0.097 | 0.614 | 2.93 |
| Guinea | 0.069 | -0.195 | 0.333 | 3.98 |
| Haiti | -0.01 | -0.255 | 0.236 | 4.23 |
| Liberia | -0.176 | -0.631 | 0.279 | 2.13 |
| Malawi | -0.407 | -0.711 | -0.102 | 3.47 |
| Maldives | 0.219 | -0.166 | 0.604 | 2.66 |
| Mali | 0.323 | 0.158 | 0.487 | 5.44 |
| Nepal | 0.166 | -0.153 | 0.485 | 3.3 |
| Nigeria | 0.283 | 0.114 | 0.453 | 5.36 |
| Pakistan | 0.156 | -0.193 | 0.505 | 2.99 |
| Papua New Guinea | -0.002 | -0.395 | 0.391 | 2.59 |
| Rwanda | -0.199 | -0.493 | 0.096 | 3.59 |
| Senegal | -0.012 | -0.263 | 0.238 | 4.16 |
| Sierra Leone | 0.258 | -0.059 | 0.574 | 3.34 |
| South Africa | 0.217 | -0.415 | 0.85 | 1.29 |
| Tajikistan | -0.1 | -0.372 | 0.172 | 3.87 |
| Tanzania | 0.153 | -0.074 | 0.379 | 4.5 |
| Timor-Leste | 0.025 | -0.145 | 0.196 | 5.35 |
| Uganda | -0.167 | -0.45 | 0.116 | 3.73 |
| Zambia | -0.17 | -0.384 | 0.045 | 4.67 |
| Zimbabwe | -0.093 | -0.333 | 0.146 | 4.3 |
| **Overall (p=0.252)** | **0.046** | **-0.033** | **0.126** | **100** |
| **Heterogeneity Measures** | **Value** | **df** | | **p-value** |
| Cochran's Q | 61.42 | 26 | | 0.000 |
|  | | **95% Conf. Interval** | |  |
| H | 1.537 | 1.096 | 1.977 |  |
| I2 (%) | 57.7% | 16.8% | 74.4% |  |

Table S 3: Unadjusted Risk Differences for Child Morbidity Between Wasted and Nonwasted Children, by Country

| **Country** | **Risk Difference** | **95% Conf. Interval** | | **% Weight** |
| --- | --- | --- | --- | --- |
| Albania | -2.312 | -3.762 | -0.862 | 0.3 |
| Angola | 0.398 | 0.077 | 0.719 | 3.99 |
| Armenia | 0.345 | -0.294 | 0.984 | 1.38 |
| Benin | 0.464 | 0.277 | 0.651 | 6.92 |
| Burundi | 0.665 | 0.378 | 0.953 | 4.57 |
| Cameroon | 0.252 | -0.154 | 0.657 | 2.88 |
| Gambia | 0.227 | -0.204 | 0.658 | 2.64 |
| Guinea | 0.333 | 0.002 | 0.663 | 3.84 |
| Haiti | 0.205 | -0.104 | 0.514 | 4.18 |
| Liberia | 0.443 | -0.163 | 1.049 | 1.51 |
| Malawi | 0.569 | 0.164 | 0.973 | 2.89 |
| Maldives | 0.294 | -0.177 | 0.765 | 2.29 |
| Mali | 0.52 | 0.349 | 0.69 | 7.39 |
| Nepal | 0.271 | -0.05 | 0.593 | 3.99 |
| Nigeria | 0.57 | 0.386 | 0.754 | 7 |
| Pakistan | 0.358 | -0.024 | 0.74 | 3.14 |
| Papua New Guinea | 0.217 | -0.212 | 0.646 | 2.65 |
| Rwanda | 0.767 | 0.101 | 1.433 | 1.28 |
| Senegal | 0.103 | -0.158 | 0.364 | 5.1 |
| Sierra Leone | 0.46 | 0.078 | 0.843 | 3.14 |
| South Africa | 0.343 | -0.791 | 1.476 | 0.48 |
| Tajikistan | 0.387 | 0.085 | 0.688 | 4.31 |
| Tanzania | 0.468 | 0.197 | 0.739 | 4.88 |
| Timor-Leste | 0.069 | -0.102 | 0.241 | 7.36 |
| Uganda | 0.249 | -0.094 | 0.592 | 3.65 |
| Zambia | 0.164 | -0.145 | 0.473 | 4.19 |
| Zimbabwe | 0.3 | -0.018 | 0.617 | 4.05 |
| **Overall (p=0.000)** | **0.351** | **0.271** | **0.431** | **100** |
| **Heterogeneity Measures** | **Value** | **df** | | **p-value** |
| Cochran's Q | 49.73 | 26 | | 0.003 |
|  | | **95% Conf. Interval** | |  |
| H | 1.383 | 1.000 | 1.784 |  |
| I2 (%) | 47.7% | 0.0% | 68.6% |  |

Table S 4: Adjusted Risk Differences for Child Morbidity Between Wasted and Nonwasted Children, by Country^[[2]](#footnote-2)^

| **Country** | **Risk Difference** | **95% Conf. Interval** | | **% Weight** |
| --- | --- | --- | --- | --- |
| Albania | -2.493 | -3.991 | -0.994 | 0.25 |
| Angola | 0.274 | -0.06 | 0.607 | 3.83 |
| Armenia | 0.22 | -0.403 | 0.842 | 1.36 |
| Benin | 0.424 | 0.231 | 0.617 | 7.46 |
| Burundi | 0.502 | 0.201 | 0.803 | 4.43 |
| Cameroon | 0.35 | -0.055 | 0.755 | 2.83 |
| Gambia | 0.303 | -0.106 | 0.712 | 2.79 |
| Guinea | 0.262 | -0.085 | 0.608 | 3.61 |
| Haiti | 0.088 | -0.218 | 0.394 | 4.32 |
| Liberia | 0.438 | -0.141 | 1.018 | 1.54 |
| Malawi | 0.515 | 0.078 | 0.953 | 2.5 |
| Maldives | 0.258 | -0.219 | 0.734 | 2.17 |
| Mali | 0.455 | 0.281 | 0.628 | 8.24 |
| Nepal | 0.211 | -0.124 | 0.546 | 3.81 |
| Nigeria | 0.422 | 0.234 | 0.61 | 7.66 |
| Pakistan | 0.247 | -0.162 | 0.657 | 2.78 |
| Papua New Guinea | -0.036 | -0.528 | 0.456 | 2.05 |
| Rwanda | 0.626 | -0.019 | 1.271 | 1.27 |
| Senegal | 0.207 | -0.053 | 0.467 | 5.36 |
| Sierra Leone | 0.495 | 0.11 | 0.881 | 3.07 |
| South Africa | -0.032 | -1.454 | 1.39 | 0.28 |
| Tajikistan | 0.138 | -0.188 | 0.463 | 3.97 |
| Tanzania | 0.498 | 0.207 | 0.789 | 4.64 |
| Timor-Leste | 0.058 | -0.118 | 0.235 | 8.11 |
| Uganda | 0.012 | -0.336 | 0.361 | 3.58 |
| Zambia | 0.139 | -0.181 | 0.459 | 4.06 |
| Zimbabwe | 0.157 | -0.165 | 0.478 | 4.03 |
| **Overall (p=0.000)** | **0.282** | **0.205** | **0.358** | **100** |
| **Heterogeneity Measures** | **Value** | **df** | | **p-value** |
| Cochran's Q | 43.55 | 26 | | 0.017 |
|  | | **95% Conf. Interval** | |  |
| H | 1.294 | 1.000 | 1.663 |  |
| I2 (%) | 40.3% | 0.0% | 63.8% |  |

Table S 5: Unadjusted Risk Differences for Child Morbidity Between Overweight and Nonoverweight Children, by Country

| **Country** | **Risk Difference** | **95% Conf. Interval** | | **% Weight** |
| --- | --- | --- | --- | --- |
| Albania | -0.149 | -0.596 | 0.298 | 3.22 |
| Angola | -0.135 | -0.635 | 0.364 | 2.72 |
| Armenia | 0.022 | -0.456 | 0.499 | 2.92 |
| Benin | -0.001 | -0.315 | 0.312 | 5.16 |
| Burundi | -0.209 | -0.681 | 0.263 | 2.97 |
| Cameroon | -0.416 | -0.679 | -0.153 | 6.22 |
| Gambia | 0.474 | -0.119 | 1.068 | 2.06 |
| Guinea | -0.167 | -0.528 | 0.194 | 4.33 |
| Haiti | -0.039 | -0.379 | 0.301 | 4.68 |
| Liberia | -0.466 | -0.996 | 0.064 | 2.48 |
| Malawi | -0.83 | -1.173 | -0.488 | 4.63 |
| Maldives | 0.085 | -0.493 | 0.664 | 2.15 |
| Mali | -0.368 | -0.785 | 0.049 | 3.57 |
| Nepal | -0.066 | -1.006 | 0.873 | 0.92 |
| Nigeria | -0.231 | -0.591 | 0.128 | 4.36 |
| Pakistan | -0.047 | -0.545 | 0.45 | 2.74 |
| Papua New Guinea | -0.135 | -0.55 | 0.279 | 3.6 |
| Rwanda | -0.379 | -0.719 | -0.039 | 4.68 |
| Senegal | -0.254 | -0.853 | 0.346 | 2.03 |
| Sierra Leone | -0.104 | -0.525 | 0.318 | 3.51 |
| South Africa | 0.262 | -0.214 | 0.739 | 2.93 |
| Tajikistan | -0.618 | -1.125 | -0.112 | 2.66 |
| Tanzania | -0.251 | -0.567 | 0.064 | 5.11 |
| Timor-Leste | 0.01 | -0.359 | 0.38 | 4.2 |
| Uganda | -0.188 | -0.557 | 0.181 | 4.22 |
| Zambia | -0.228 | -0.484 | 0.028 | 6.39 |
| Zimbabwe | -0.014 | -0.309 | 0.281 | 5.53 |
| **Overall (p=0.000)** | **-0.188** | **-0.282** | **-0.095** | **100** |
| **Heterogeneity Measures** | **Value** | **df** | | **p-value** |
| Cochran's Q | 37.48 | 26 | | 0.068 |
|  | | **95% Conf. Interval** | |  |
| H | 1.201 | 1.000 | 1.528 |  |
| I2 (%) | 30.6% | 0.0% | 57.2% |  |

Table S 6: Adjusted Risk Differences for Child Morbidity Between Overweight and Nonoverweight Children, by Country^[[3]](#footnote-3)^

| **Country** | **Risk Difference** | **95% Conf. Interval** | | **% Weight** |
| --- | --- | --- | --- | --- |
| Albania | -0.144 | -0.599 | 0.312 | 3.34 |
| Angola | -0.302 | -0.819 | 0.215 | 2.72 |
| Armenia | -0.187 | -0.692 | 0.319 | 2.82 |
| Benin | -0.115 | -0.442 | 0.212 | 5.43 |
| Burundi | -0.248 | -0.749 | 0.252 | 2.87 |
| Cameroon | -0.394 | -0.676 | -0.111 | 6.54 |
| Gambia | 0.141 | -0.468 | 0.75 | 2.05 |
| Guinea | -0.25 | -0.63 | 0.129 | 4.42 |
| Haiti | -0.112 | -0.459 | 0.235 | 5 |
| Liberia | -0.743 | -1.307 | -0.178 | 2.34 |
| Malawi | -1.064 | -1.454 | -0.674 | 4.24 |
| Maldives | 0.083 | -0.542 | 0.708 | 1.96 |
| Mali | -0.386 | -0.808 | 0.036 | 3.76 |
| Nepal | -0.187 | -1.147 | 0.772 | 0.9 |
| Nigeria | -0.254 | -0.61 | 0.102 | 4.83 |
| Pakistan | -0.099 | -0.633 | 0.435 | 2.58 |
| Papua New Guinea | 0.034 | -0.517 | 0.586 | 2.44 |
| Rwanda | -0.413 | -0.76 | -0.065 | 4.99 |
| Senegal | -0.702 | -1.323 | -0.081 | 1.98 |
| Sierra Leone | -0.09 | -0.516 | 0.337 | 3.7 |
| South Africa | 0.263 | -0.407 | 0.933 | 1.74 |
| Tajikistan | -0.626 | -1.152 | -0.1 | 2.64 |
| Tanzania | -0.367 | -0.691 | -0.044 | 5.51 |
| Timor-Leste | -0.104 | -0.481 | 0.273 | 4.45 |
| Uganda | -0.317 | -0.709 | 0.075 | 4.21 |
| Zambia | -0.42 | -0.695 | -0.146 | 6.75 |
| Zimbabwe | -0.245 | -0.556 | 0.066 | 5.79 |
| **Overall (p=0.000)** | **-0.293** | **-0.386** | **-0.199** | **100** |
| **Heterogeneity Measures** | **Value** | **df** | | **p-value** |
| Cochran's Q | 35.50 | 26 | | 0.101 |
|  | | **95% Conf. Interval** | |  |
| H | 1.169 | 1.000 | 1.487 |  |
| I2 (%) | 26.8% | 0.0% | 54.8% |  |

Table S 7: Effects of Childhood Malnutrition on Morbidity: A Comparative Analysis Using Propensity Score Matching

| **Form of Malnutrition** | **Exposed (Risk)** | **Unexposed (Risk)** | **Total (Risk)** | **Risk Ratio** | **95% Conf. Interval** | | **p-value** |
| --- | --- | --- | --- | --- | --- | --- | --- |
| Double Burden | 0.299 | 0.304 | 0.303 | 0.983 | 0.95 | 1.016 | 0.31 |
| Wasting | 0.332 | 0.303 | 0.309 | 1.094 | 1.053 | 1.136 | <0.001 |
| Overweight | 0.244 | 0.304 | 0.293 | 0.800 | 0.758 | 0.846 | <0.001 |

Table S 8: Population Attributable Fraction of Double Burden Malnutrition in Child Morbidity

| **Country** | **Estimate** | **Minimum** | **Maximum** |
| --- | --- | --- | --- |
| Benin | 0.024 | 0.004 | 0.044 |
| Burundi | 0.018 | 0.001 | 0.035 |
| Mali | 0.042 | 0.014 | 0.070 |
| Nigeria | 0.030 | 0.008 | 0.052 |
| **Overall** | **0.028** | **0.017** | **0.039** |

Table S 9: Population Attributable Fraction of Wasting in Child Morbidity

| **Country** | **Estimate** | **Minimum** | **Maximum** |
| --- | --- | --- | --- |
| Benin | 0.027 | 0.004 | 0.050 |
| Burundi | 0.034 | 0.013 | 0.054 |
| Malawi | 0.015 | -0.007 | 0.037 |
| Mali | 0.073 | 0.040 | 0.105 |
| Nigeria | 0.046 | 0.022 | 0.070 |
| Sierra Leone | 0.076 | 0.015 | 0.135 |
| Tanzania | 0.015 | -0.021 | 0.049 |
| **Overall** | **0.037** | **0.026** | **0.048** |

1. Adjusted for Child’s age, Child’s sex, Place of residence, Childbirth type, breastfeeding status, maternal age, maternal education, maternal marital status, maternal employment, wealth index, and maternal health behavior [↑](#footnote-ref-1)
2. Adjusted for Child’s age, Child’s sex, Place of residence, Childbirth type, breastfeeding status, maternal age, maternal education, maternal marital status, maternal employment, wealth index, and maternal health behavior [↑](#footnote-ref-2)
3. Adjusted for Child’s age, Child’s sex, Place of residence, Childbirth type, breastfeeding status, maternal age, maternal education, maternal marital status, maternal employment, wealth index, and maternal health behavior [↑](#footnote-ref-3)
